# A Few Points in the Treatment of Morphinism

**Published:** 1896-09

**Authors:** C. C. Stockard

**Affiliations:** Atlanta, Ga.


### A FEW POINTS IN THE TREATMENT OF MORPHINISM.

By C. C. STOCKARD, M.D.,

Atlanta, Ga.

The treatment of the morphine habit like that of other diseases, to be successful, requires the mixing of the remedies with brains. Each patient must be carefully studied as to inherited and acquired peculiarities, and particularly as to the trouble which originally led to the use of morphine.

If this can be removed by surgical operation or otherwise the chances of success will be greatly increased. Where this cannot be done, one must expect probably the greatest difficulty to be encountered from a return of that trouble, whether diarrhea, neuralgia or what not, and such measures should be adopted as are best calculated to limit that particular symptom.

In the large majority of cases a good hepatic stimulant and purge will do much to decrease the symptoms, and it may be necessary to repeat this as well as to follow it with intestinal antiseptics, for the heavily coated tongue, foul breath and horribly offensive stools show the need of this sort of medication, which does much to prevent and relieve vomiting, pains, and diarrhea. As to the choice of antiseptics this will depend upon the case, whether the trouble is in the bowel or in the stomach, whether there is vomiting, diarrhea, or constipation.

A very important point too in the limiting of alimentary troubles is the diet. Many patients have good appetites during the withdrawal of the opiate and will eat any and everything if allowed to, but will suffer evil consequences afterwards. The diet should be simple and nourishing, and those things likely to increase fermentative processes avoided.

With what is known as the *rapid* method, which is between the *sudden* withdrawal of Levinstein and the *slow* method, serious collapse is not likely to occur, but when the patient is debilitated to begin with, and, especially if there are vomiting and diarrhea, it may appear, and must be looked for and promptly met.

This rapid method is the one most frequently adopted, as few patients are willing to undergo the tortures of the sudden method, and few physicians to subject patients to the unnecessary suffering and the danger involved. The slow method has not proved successful, and the suffering it entails is too prolonged.

The successful management of these cases requires, on the part of the physician, firmness moderated by kindness, and a good knowledge of medicine in general; thoroughly trustworthy nurses, and constant vigilance on the part of both physician and nurses.

The morphinist undergoing treatment is constantly doing the unexpected. I thwarted a patient three times in one day in attempts at suicide. In one of these, being left in the room alone for a few moments, he detached the tube from a gas stove, turned on the gas and went to bed, putting the tube in his mouth.

Of course, above everything else, it is important that the physician know that his patient gets only what he directs, and one cannot be sure of this when treating a patient at home or among friends, for they will not resist the piteous appeals almost sure to be made.

The actual suffering in the majority of well managed cases is not great, but as the morphine is eliminated from the system a supersensitiveness develops, so that what an ordinary person would hardly complain of, to the morphinist is "suffering the tortures of the damned."

The time passes slowly for these patients, and much in the way of pleasant diversion is needed, but this must not be at the expense of giving the patient opportunity for obtaining morphine.

---

A HEAPING tablespoonful of washing soda to a quart of water is the proper proportion for the solution in which instruments should be boiled for sterilization. *Do not* boil non-metallic sutures in this liquid, for it will very greatly weaken them. *Do not* boil an aluminum instrument in this liquid, for it will be corroded and completely ruined. Silk sutures and aluminum instruments may be sterilized by boiling in five per cent. carbolic.—*International Journal of Surgery.*

